# A Constrained Kalman Filter for Wi-Fi-Based Indoor Localization with Flexible Space Organization

**DOI:** 10.3390/s22020428

**Published:** 2022-01-06

**Authors:** Vincent Sircoulomb, Houcine Chafouk

**Affiliations:** IRSEEM, ESIGELEC, UNIROUEN, Normandie University, 76000 Rouen, France; houcine.chafouk@esigelec.fr

**Keywords:** constrained Kalman filtering, Wi-Fi, Localization Based Service (LBS), flexible indoor organization

## Abstract

This paper presents a constrained Kalman filter for Wi-Fi-based indoor localization. The contribution of this work is to introduce constraints on the object speed and to provide a numerically optimized form for fast computation. The proposed approach is suitable to flexible space organization, as in warehouses, and when objects can be spun around, for example barcode readers in a hand. We experimented with the proposed technique using a robot and three devices, on five different journeys, in a 6000 m^2^ warehouse equipped with six Wi-Fi access points. The results highlight that the proposed approach provides a 19% improvement in localization accuracy.

## 1. Introduction

Nowadays, a wide-scale proliferation of wireless devices can be observed. This phenomenon is due to multiple factors, such as more and more efficient, small and low energy consumption electronic components and widespread wireless communication technologies. Among the services allowed by this technological progress, LBS (Localization Based Service) is facing a continuously increasing interest, notably due to dedicated smartphone applications. According to the statistics, 80% of LBS are for indoor purposes [1], for example for guidance of persons with mobility problems, emergency evacuation or goods localization in warehouses.

In the past decade, plenty of research has been conducted about LBS [2,3], in particular using wireless techniques [4,5,6] such as ultra-wideband, Bluetooth, or Wi-Fi. Wi-Fi has the advantage to be cost-advantageous. Nowadays, it is also very widespread, which avoids the installation of new antennas or additional transponders as for RFID (Radio Frequency IDentification) for example. Position information can be obtained through the wireless signal RSSI (Received Signal Strength Indication) [7] or the waves’ AoA (Angle of Arrival) [8], knowing the position of the emitters. However, such information is generally insufficient to provide accurate localization, especially using Wi-Fi. Thus, wireless localization is traditionally coupled to additional techniques such as fingerprinting [9]; the use of space grids [10,11]; or of additional sensors like smartphone ones, namely accelerometers, gyroscopes, or magnetometers [12]. This last technique is usually named inertial navigation and includes a variant called PDR (Pedestrian Dead Reckoning).

To fuse information coming from Wi-Fi and from one or several additional technique(s), Kalman filtering is the most popular approach [13]. Many improvements to the baseline Kalman Filter (KF) have been used, such as the extended (EKF) or unscented KF (UKF) to deal with the orientation nonlinearity, multi-stage, and adaptive or robust versions. In [14], landmarks were used as inputs of a limited complexity KF to improve the accuracy. In [15], two EKF were used to fuse the measurements and to update a fingerprinting database. In [16], an adaptive UKF was used to improve the PDR with a map-matching technique. In [17], particle swarm optimization was used to improve the correction step accuracy. In [18], several movement scenarios were considered with an adaptive and robust filter. In [19], a two-stage version was introduced to improve the robustness by separately dealing with attitude determination and tracking.

Another improvement in KF is state constraints. State constraints are all additional equations that do not fit into the structure of a KF [20]. For example, when estimating a quaternion, it is known that its Euclidian norm is equal to one. Taking into account such additional knowledge in the estimation process contributes to improving the localization and tracking accuracy. 

### 1.1. Related Work

Extensive research has been conducted to incorporate state constraint equations into the structure of a state estimator—through model reparameterization or model reduction, pseudo-observation methods, gain modification techniques, projection approaches, etc. If both the system and constraints are linear, all these approaches result in the same state estimate [21]. For LBS purposes, constraints about the heading were taken into account in [1,22], allowing for a reduction of the state space-tracking model to a four-order linear one. In [23], the heading was constrained according to the type of environment: narrow passages like corridors versus wide scenes with an azimuth wheel. In [24], position constraints were considered in an UKF when at most four anchors contributed to the localization. In [25], nonlinear inequality constraints were considered for the measurements, in particular RSSI and AoA. However, it should be noted that no work has been done to consider speed constraints in KF-based LBS.

### 1.2. Contribution

In this paper, the contribution is to develop a Kalman filter for Wi-Fi-based indoor LBS with constraints on the moving object speed. This contribution arises from the kind of indoor environment under consideration in this work: a warehouse with flexible topology and space organization, according to the products stored. The nature of the products can badly affect Wi-Fi coverage, impacting localization. Moreover, small IMU chips with a limited cost usually have larger drifts and errors, which do not contribute to improving the positioning. State constraints are thus considered to compensate for this accuracy decrease. The flexible topology implies that the paths can evolve with time, making position constraints unsuitable. Moreover, the objects to localize can be spun around anywhere, preventing the consideration of heading constraints. We thus introduce speed constraints according to the limited knowledge we have about the flexible organization. A corresponding Kalman LBS algorithm is developed in this paper and the constraint stage is detailed to provide fast computation with a minimal amount of scalar additions and multiplications.

### 1.3. Outline

This paper is organized as follows. The problem to solve is first stated in Section 2. Then, the proposed approach to deal with the problem under consideration is depicted in Section 3. The results obtained on several practical case studies in a warehouse are given in terms of plots and figures in Section 4. These results are then analyzed and discussed in Section 5. Finally, concluding remarks and the outlook of this work are given in Section 6.

## 2. Problem Statement

**Figure 1:**
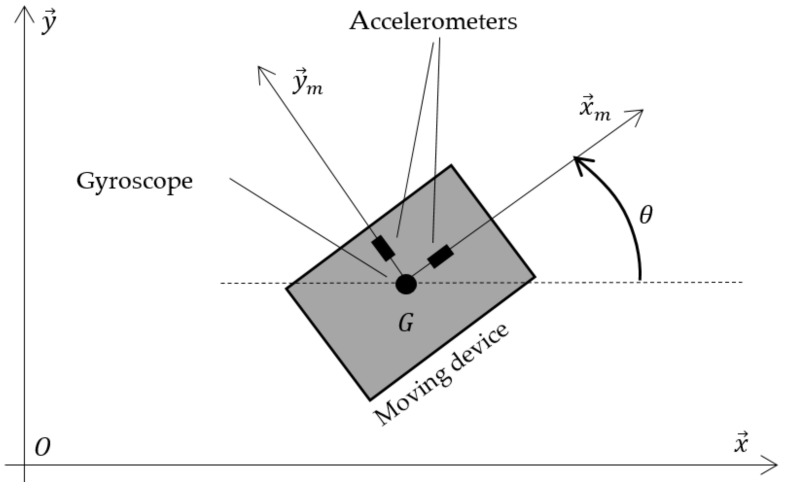
Moving device to track into an indoor environment.

Let us consider a device moving into an indoor environment. The movement is supposed to be horizontal. The device is equipped with an IMU (Inertial Measurement Unit). The objective is to track the device, that is to say to estimate its position in real time. The device movement can be modeled by Equation (1).

(1) $$\left[\begin{array}{c} r_{x,k}\\ r_{y,k}\\ V_{x,k}\\ V_{y,k}\\ \theta_{k} \end{array}\right]=\left[\begin{array}{ccccc} 1 & 0 & T_{s} & 0 & 0\\ 0 & 1 & 0 & T_{s} & 0\\ 0 & 0 & 1 & 0 & 0\\ 0 & 0 & 0 & 1 & 0\\ 0 & 0 & 0 & 0 & 1 \end{array}\right]\left[\begin{array}{c} r_{x,k-1}\\ r_{y,k-1}\\ V_{x,k-1}\\ V_{y,k-1}\\ \theta_{k-1} \end{array}\right]+\left[\begin{array}{ccc} 0 & 0 & 0\\ 0 & 0 & 0\\ \cos\left(\theta_{k-1}\right) & -\sin\left(\theta_{k-1}\right) & 0\\ \sin\left(\theta_{k-1}\right) & \cos\left(\theta_{k-1}\right) & 0\\ 0 & 0 & 1 \end{array}\right]\left[\begin{array}{c} \Delta V_{x,k}\\ \Delta V_{y,k}\\ \Delta \theta_{k} \end{array}\right],$$

where $k\in {\mathbb{N}}^{*}$ is the discrete time; $T_{s}$ is the sampling time; $\left(r_{x,k};r_{y,k}\right)$ and $\left(V_{x,k};V_{y,k}\right)$ denote the 2D position and velocity, respectively, at time k; and where $\theta_{k}$ is the device heading (see Figure 1). $\Delta V_{x,k}$, $\Delta V_{y,k}$, and $\Delta \theta_{k}$ are the IMU measurements, that is to say the velocity increments between discrete times k−1 and k along the $\left(G,{\vec{x}}_{m}\right)$ and $\left(G,{\vec{y}}_{m}\right)$ axes and the angle increment around the $\left(G,\;\vec{z}\right)$ axis. Writing down on the first hand:

(2) $$x=\left[\begin{array}{c} r_{x}\\ r_{y}\\ V_{x}\\ V_{y}\\ \theta \end{array}\right],\;\;u=\left[\begin{array}{c} \Delta V_{x}\\ \Delta V_{y}\\ \Delta \theta \end{array}\right],\;\;F=\left[\begin{array}{ccccc} 1 & 0 & T_{s} & 0 & 0\\ 0 & 1 & 0 & T_{s} & 0\\ 0 & 0 & 1 & 0 & 0\\ 0 & 0 & 0 & 1 & 0\\ 0 & 0 & 0 & 0 & 1 \end{array}\right],\;\;G\left(x\right)=\left[\begin{array}{ccc} 0 & 0 & 0\\ 0 & 0 & 0\\ \cos\left(\theta \right) & -\sin\left(\theta \right) & 0\\ \sin\left(\theta \right) & \cos\left(\theta \right) & 0\\ 0 & 0 & 1 \end{array}\right],$$

and considering process noises in the second-hand, Equation (1) can be rewritten as follows:

(3) $$x_{k}=Fx_{k-1}+G\left(x_{k-1}\right)\left(u_{k}+s_{k}\right)+w_{k}.$$

where $s_{k}$ is the additive noise affecting the IMU measurements and $w_{k}$ is introduced to take into account the model approximations (discretization, neglecting the earth rotation effect, etc.).

To compensate for the mid- and long-term effects of the stochastic processes $\left\{s_{k}\right\}$ and $\left\{w_{k}\right\}$, the device position is also measured according to Equation (4):

(4) $${\mathrm{RSSI}}_{i}=-10n\log_{10}\left(d_{i}\right)+A_{i},$$

where ${\mathrm{RSSI}}_{i}$ is the Received Signal Strength Indication of the Wi-Fi signals exchanged with the access point $i\;\left(1\leq i\leq N\right)$, n is a factor depending on the environment (n=2 for outdoor and 4 for indoor situations), $d_{i}$ is the distance between the device and the access point, and $A_{i}$ is a calibration coefficient. Introducing the following:

(5) $$y_{k}=\left[\begin{array}{c} {\mathrm{RSSI}}_{1,k}\\ \vdots \\ {\mathrm{RSSI}}_{N,k} \end{array}\right],\;\;\gamma_{k}=\left[\begin{array}{ccc} \gamma_{1,k} & 0 & 0\\ 0 & \ddots & 0\\ 0 & 0 & \gamma_{N,k} \end{array}\right]\;\;\mathrm{with}:\;\gamma_{i,k}=\left\{\begin{array}{l} 1\;\mathrm{if}\;{\mathrm{RSSI}}_{i}\;\mathrm{is}\;\mathrm{detected}\;\mathrm{at}\;\mathrm{time}\;k\\ 0\;\mathrm{otherwise} \end{array}\right.$$

Equation (4) can be rewritten in the following compact form:

(6) $$y_{k}=\gamma_{k}\left(h\left(x_{k}\right)+v_{k}\right),$$

where $v_{k}$ is the measurement noise. The problem to solve is to estimate as accurately as possible $x_{k}$ for any time k using the set of measurements $\left\{y_{1},\ldots ,y_{k}\right\}$ and their existence $\left\{\gamma_{1},\ldots ,\gamma_{k}\right\}$.

Using Equations (3) and (6), the estimation can recursively be done using an EKF, because function h is easy to linearize and does not contain discontinuities. The EKF Equations (7)–(8) for the prediction step and Equations (9)–(11) for the Joseph’s form correction step are given below:

(7) $${\hat{x}}_{k}^{-}=F{\hat{x}}_{k-1}+G\left({\hat{x}}_{k-1}\right)u_{k},$$

(8) $$\Sigma_{k}^{-}=F\Sigma_{k-1}F^{T}+G\left({\hat{x}}_{k-1}\right)PG^{T}\left({\hat{x}}_{k-1}\right)+Q,$$

(9) $$K_{k}=\gamma_{k}\Sigma_{k}^{-}H_{k}^{T}{\left(H_{k}\Sigma_{k}^{-}H_{k}^{T}+R\right)}^{-1},$$

(10) $${\hat{x}}_{k}^{+}={\hat{x}}_{k}^{-}+K_{k}\left(y_{k}-h\left({\hat{x}}_{k}^{-}\right)\right)$$

(11) $$\Sigma_{k}^{+}=\left(I-K_{k}H_{k}\right)\Sigma_{k}^{-}{\left(I-K_{k}H_{k}\right)}^{T}+K_{k}RK_{k}^{T}$$

where I is the identity matrix of appropriate dimension; the superscript T denotes the transposition operation; and $P,Q,\;R,\Sigma_{k}^{-}$, and $\Sigma_{k}^{+}$ are the following covariances:

(12) $$\begin{array}{c} P=E\left(s_{k}s_{k}^{T}\right),\;\;\;\;\;Q=E\left(w_{k}w_{k}^{T}\right),\;\;\;\;\;R=E\left(v_{k}v_{k}^{T}\right),\\ \Sigma_{k}^{-}=E\left(\left(x_{k}-{\hat{x}}_{k}^{-}\right){\left(x_{k}-{\hat{x}}_{k}^{-}\right)}^{T}\right),\;\;\;\Sigma_{k}^{+}=E\left(\left(x_{k}-{\hat{x}}_{k}^{+}\right){\left(x_{k}-{\hat{x}}_{k}^{+}\right)}^{T}\right). \end{array}$$

The filter is initialized with ${\hat{x}}_{0}$ and $\Sigma_{0}$. It should be noted that at a given time k, if no RSSI is detected, then $\gamma_{k}$ is null and so ${\hat{x}}_{k}^{+}={\hat{x}}_{k}^{-}$ and $\Sigma_{k}^{+}=\Sigma_{k}^{-}$. As output of the EKF, the estimated state and error covariance are as follows: ${\hat{x}}_{k}≜{\hat{x}}_{k}^{+}$ and $\Sigma_{k}≜\Sigma_{k}^{+}$. The matrix $H_{k}$ is obtained by linearizing h, as follows:

(13) $$H_{k}={\left.\frac{\partial h}{\partial x}\right|}_{x={\hat{x}}_{k}^{-}}=-\frac{10n}{\log_{2}\left(10\right)}\left[\begin{array}{ccccc} \frac{{\hat{x}}_{k}^{-}\left(1\right)-X_{b1}}{d_{1}} & \frac{{\hat{x}}_{k}^{-}\left(2\right)-Y_{b1}}{d_{1}} & 0 & 0 & 0\\ \vdots & \vdots & \vdots & \vdots & \vdots \\ \frac{{\hat{x}}_{k}^{-}\left(1\right)-X_{bN}}{d_{N}} & \frac{{\hat{x}}_{k}^{-}\left(2\right)-Y_{bN}}{d_{N}} & 0 & 0 & 0 \end{array}\right],$$

where $\left(X_{bi};Y_{bi}\right)$ are the coordinates of the access point i  and where $d_{i}=\sqrt{{\left({\hat{x}}_{k}^{-}\left(1\right)-X_{bi}\right)}^{2}+{\left({\hat{x}}_{k}^{-}\left(2\right)-Y_{bi}\right)}^{2}}$ is the distance between the object predicted position $\left({\hat{x}}_{k}^{-}\left(1\right);{\hat{x}}_{k}^{-}\left(2\right)\right)$ and the access point i.

Localizations using Wi-Fi RSSI, IMU, and EKF usually give pretty good results, with the accuracy depending on the number of access points. However, in warehouses, the Wi-Fi coverage is often depreciated because of metallic shelving and harsh stored products such as pallets of water bottles, conserves, or chemical products. Moreover, the topology of warehouses can frequently change because of permanently changing stocks. Thus, additional state constraints are introduced in the baseline EKF to improve the localization accuracy. 

## 3. Proposed Approach

**Figure 2:**
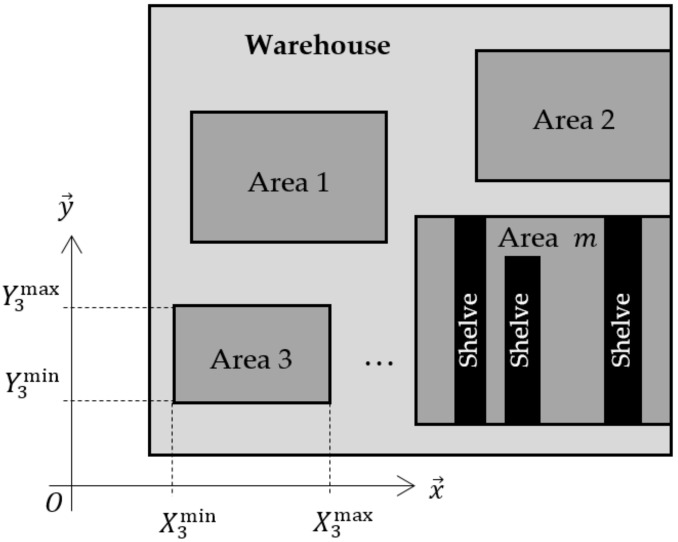
Varying organization of the warehouse, with m storage areas. An example of shelves’ organization is drawn for area m.

Let us consider an indoor environment such as a warehouse. It includes several rectangular storage areas containing shelves, as illustrated in Figure 2. Each storage area can have its own organization changed, i.e., the shelves’ position and quantity can be modified according to the products stored. Consequently, the corridors between the shelves are not fixed. Outside the storage areas, the movements are free. For circulation convenience, all shelves are parallel to the $\left(O,\vec{y}\right)$ axis.

Because the shelves’ localization into an area are subject to possible modifications, position constraints are not suitable. Moreover, the objects to localize can be spun around, for example a barcode reader in a hand when scanning some products put on the shelves. Thus, no additional constraint about the heading could be taken into account. However, as the displacements between the shelves are along the $\left(O,\vec{y}\right)$ axis due to the area arrangement, the speed along the $\left(O,\vec{x}\right)$ axis is close to zero. This constraint is taken into account according to a smooth additional measurement, as in [26]:

(14) $$Dx_{k}+\zeta_{k}=0\;\;\mathrm{with}:\;D=\left[0\;0\;1\;0\;0\right].$$

Constraint (14) is equivalent to: $V_{x,k}=\zeta_{k}$, where $\left\{\zeta_{k}\right\}$ is a zero-mean white random process with a standard deviation σ. It imposes the speed along the $\left(O,\vec{x}\right)$ axis to be close to zero, but not strictly, because the object can nevertheless laterally navigate between the left and right shelves. Equation (14) must be considered only if the object is inside a storage area. Writing down $\left(X_{i}^{\min},X_{i}^{\max},Y_{i}^{\min},Y_{i}^{\max}\right)$, the limits of area i (see Figure 2), and considering the uncertainty of the position estimate $\left({\hat{x}}_{k}^{+}\left(1\right);{\hat{x}}_{k}^{+}\left(2\right)\right)$, the condition is as follows:

(15) $$\left\{\begin{array}{c} X_{i}^{\min}+3\sqrt{\Sigma_{k}^{+}\left(1,1\right)}\leq {\hat{x}}_{k}^{+}\left(1\right)\leq X_{i}^{\max}-3\sqrt{\Sigma_{k}^{+}\left(1,1\right)}\\ Y_{i}^{\min}+3\sqrt{\Sigma_{k}^{+}\left(2,2\right)}\leq {\hat{x}}_{k}^{+}\left(2\right)\leq Y_{i}^{\max}-3\sqrt{\Sigma_{k}^{+}\left(2,2\right)} \end{array}\right.,$$

where $\Sigma_{k}^{+}\left(i,j\right)$ is the coefficient at the $i^{\mathrm{th}}$ row and $j^{\mathrm{th}}$ column of $\Sigma_{k}^{+}$. Considering the noise distribution is Gaussian, the probability of wrong detection of a storage area is less than 0.1%. When condition (15) is satisfied for one of the storage areas, taking into account constraint (14) leads to the following constraint update, similar to the correction step of Equations (9)–(11):

(16) $$L_{k}=\Sigma_{k}^{+}D^{T}{\left(D\Sigma_{k}^{+}D^{T}+\sigma^{2}\right)}^{-1},$$

(17) $${\hat{x}}_{k}={\hat{x}}_{k}^{+}+L_{k}\left(0-D{\hat{x}}_{k}^{+}\right)=\left(I-L_{k}D\right){\hat{x}}_{k}^{+},$$

(18) $$\Sigma_{k}=\left(I-L_{k}D\right)\Sigma_{k}^{+},$$

where ${\hat{x}}_{k}$ and $\Sigma_{k}$ are the constrained estimated state and covariance, respectively. Considering that $D=\left[0\;0\;1\;0\;0\right]$, Equation (16) becomes the following:

(19) $$L_{k}=\frac{1}{\Sigma_{k}^{+}\left(3,3\right)+\sigma^{2}}\left[\begin{array}{c} \Sigma_{k}^{+}\left(1,3\right)\\ \vdots \\ \Sigma_{k}^{+}\left(5,3\right) \end{array}\right].$$

This result allows for writing down the following:

(20) $$M_{k}=I-L_{k}D=\left[\begin{array}{ccccc} 1 & 0 & -\alpha_{1k} & 0 & 0\\ 0 & 1 & -\alpha_{2k} & 0 & 0\\ 0 & 0 & \beta_{k} & 0 & 0\\ 0 & 0 & -\alpha_{4k} & 1 & 0\\ 0 & 0 & -\alpha_{5k} & 0 & 1 \end{array}\right]\;\;\mathrm{with}:\left\{\begin{array}{l} T_{k}=\frac{1}{\Sigma_{k}^{+}\left(3,3\right)+\sigma^{2}}\\ \alpha_{ik}=T_{k}\Sigma_{k}^{+}\left(i,3\right)\;\;i=1,2,4,5\\ \beta_{k}=T_{k}\sigma^{2} \end{array}\right.$$

Finally, the constraints in scalar form for fast computation become the following:

(21) $$\left\{\begin{array}{l} {\hat{x}}_{k}\left(i\right)={\hat{x}}_{k}^{+}\left(i\right)-\alpha_{ik}{\hat{x}}_{k}^{+}\left(3\right)\;\;i=1,2,4,5\\ {\hat{x}}_{k}\left(3\right)=\beta_{k}{\hat{x}}_{k}^{+}\left(3\right)\\ \Sigma_{k}\left(i,j\right)=\Sigma_{k}^{+}\left(i,j\right)-\alpha_{ik}\Sigma_{k}^{+}\left(3,j\right)\;\;i=1,2,4,5,\;j=1,\ldots ,5\\ \Sigma_{k}\left(3,j\right)=\beta_{k}\Sigma_{k}^{+}\left(3,j\right)\;\;\;j=1,\ldots ,5 \end{array}\right.\;\;$$

Equations (20) and (21) can be optimally computed with 25 additions (1 for Equation (20), 4 for ${\hat{x}}_{k}$, and 4 × 5 for $\Sigma_{k}$) and 36 multiplications (6 for Equation (20), 5 for ${\hat{x}}_{k}$, and 4 × 5 + 5 for $\Sigma_{k}$). 

**Figure 3:**
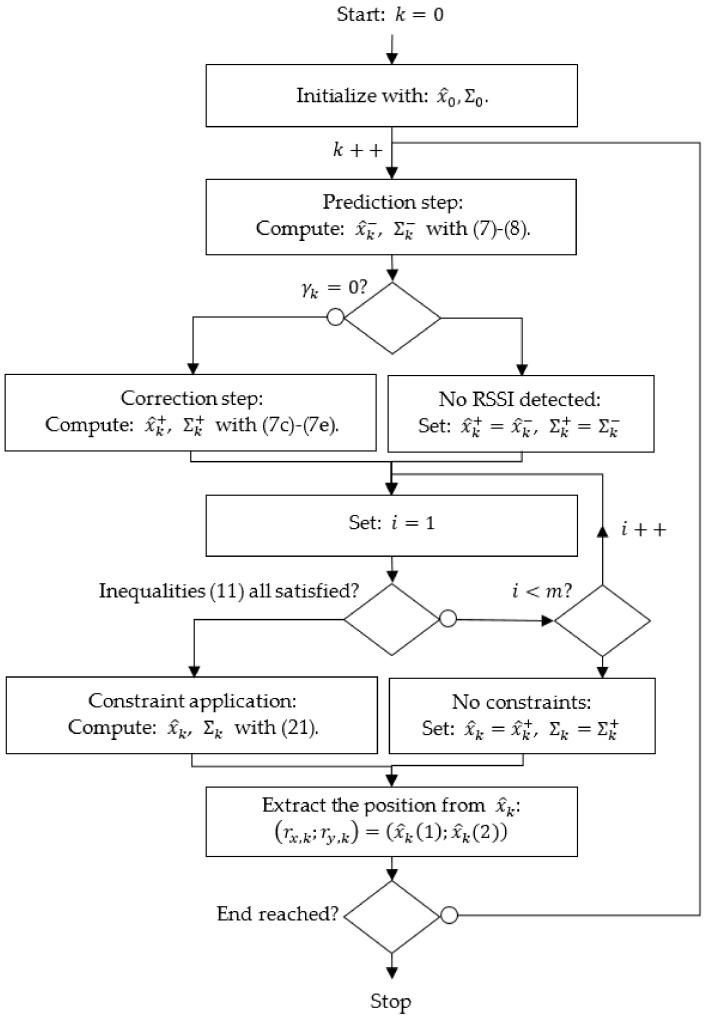
Flowchart of the proposed constrained LBS approach.

The flowchart of the constrained LBS is summarized in Figure 3. 

## 4. Results

**Figure 4:**
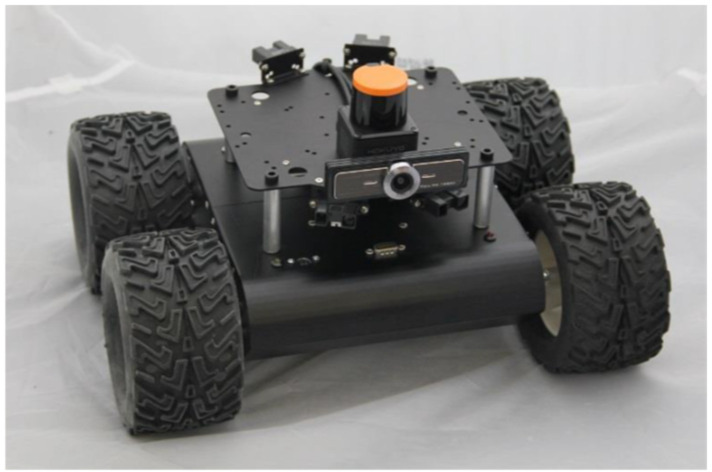
WiFibot moving into the warehouse.

**Figure 5:**
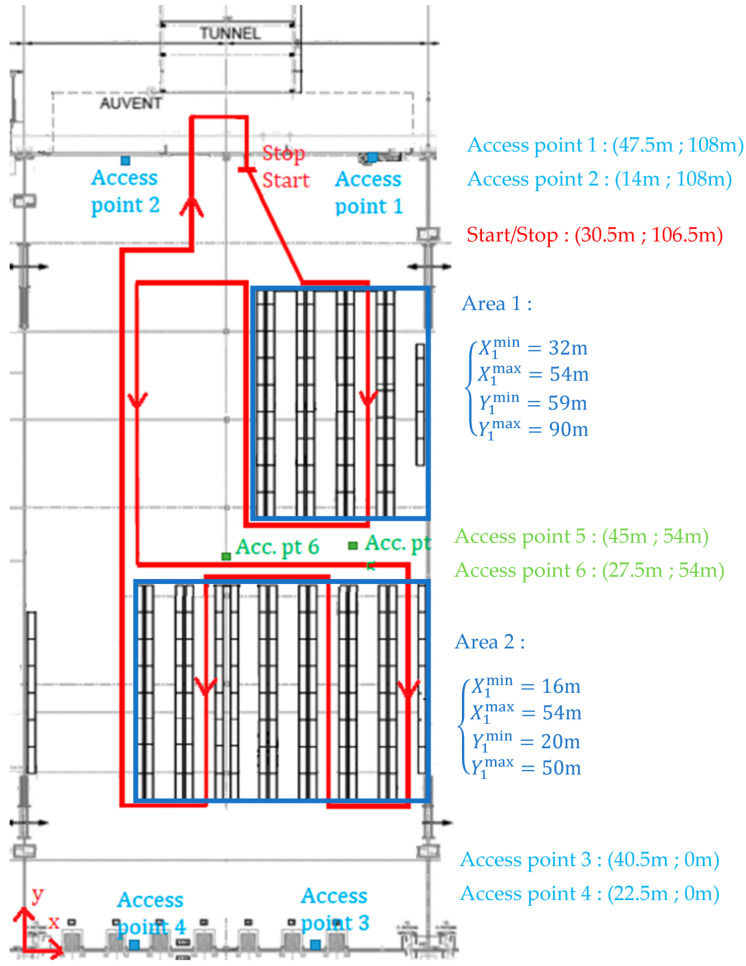
Warehouse under consideration with the 425 m journey done by the robot and the localization of the different Wi-Fi access points and storage areas.

Now, let us apply the proposed approach to a 6000 $m^{2}$ warehouse. This warehouse contains m=2 storage areas and is equipped with N=6 Wi-Fi access points (reference: Cambium CnPilot E510 with firmware 3.11.4.1-r3). A 425 m journey is done with a robot (see Figure 4), according to the route drawn in Figure 5. The robot is a WiFibot Lab V4 equipped with an Intel Celeron quad core SBC running Linux Ubuntu 18.04 LTS. It embeds for the wireless communication an Atheros AR9280 wireless-N dual band half mini-card. The robot is also equipped with a YEI 3-space IMU, whose noise characteristics are 99 $\mu g/\sqrt{\mathrm{Hz}}$ for the accelerometers and 9 $\mathrm{mdeg}/\sqrt{s}$ for the gyroscopes, leading to the following P with sample time $T_{s}=0.1\;s$:

(22) $$P=10^{-8}\left[\begin{array}{ccc} 9.424 & 0 & 0\\ 0 & 9.424 & 0\\ 0 & 0 & 0.247 \end{array}\right].\;\;$$

To set the parameters $A_{i},n\;$ and the variance R, the robot was placed at different known fixed positions all over the warehouse. About 2000 RSSI values coming from the six access points were acquired. Remarking that Equation (4) is linear in parameters $A_{i}$ and n, the least squares method can be applied to compute their value. The variances of the computed $A_{i}$ that can further be obtained are by definition equal to R. The results are n=3.6885, $R=1\;{\mathrm{dB}}^{2}$, and:

(23) $$\begin{array}{ccccccc} \text{Acc}.\text{ pt}\;i & 1 & 2 & 3 & 4 & 5 & 6\\ A_{i}\;\left(\text{dB}\right) & -11.66 & -14.04 & -17.16 & -14.52 & -12.53 & -16.43 \end{array}$$

Finally, the additional process noise and velocity constraint covariances were set to:

(24) $$Q=10^{-6}\left[\begin{array}{ccccc} 0.01 & 0 & 0 & 0 & 0\\ 0 & 0.01 & 0 & 0 & 0\\ 0 & 0 & 25 & 0 & 0\\ 0 & 0 & 0 & 25 & 0\\ 0 & 0 & 0 & 0 & 0.04 \end{array}\right],\;\;\;\sigma^{2}=1\frac{m^{2}}{s^{2}},\;\;$$

and the EKF was initialized with Equation (25).

(25) $${\hat{x}}_{0}=\left[\begin{array}{c} 30\\ 106\\ 0\\ 0\\ -\pi /3 \end{array}\right]\approx x_{0}\;\;\Sigma_{0}=\left[\begin{array}{ccccc} 3^{2} & 0 & 0 & 0 & 0\\ 0 & 3^{2} & 0 & 0 & 0\\ 0 & 0 & 0.1^{2} & 0 & 0\\ 0 & 0 & 0 & 0.1^{2} & 0\\ 0 & 0 & 0 & 0 & 5^{2} \end{array}\right].\;\;$$

**Figure 6:**
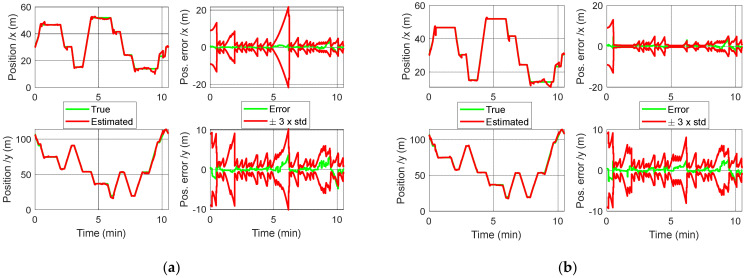
Position with respect to the time: (**a**) without application of constraints; (**b**) with application of the constraints. The right part of plots (**a**,**b**) are the error (difference between real and estimated position) and the confidence interval ±3×STD, where STD is the standard deviation: $\mathrm{STD}=\sqrt{\Sigma_{k}\left(1,1\right)}$ for the *X*-axis and $\mathrm{STD}=\sqrt{\Sigma_{k}\left(2,2\right)}$ for the *Y*-axis.

**Figure 7:**
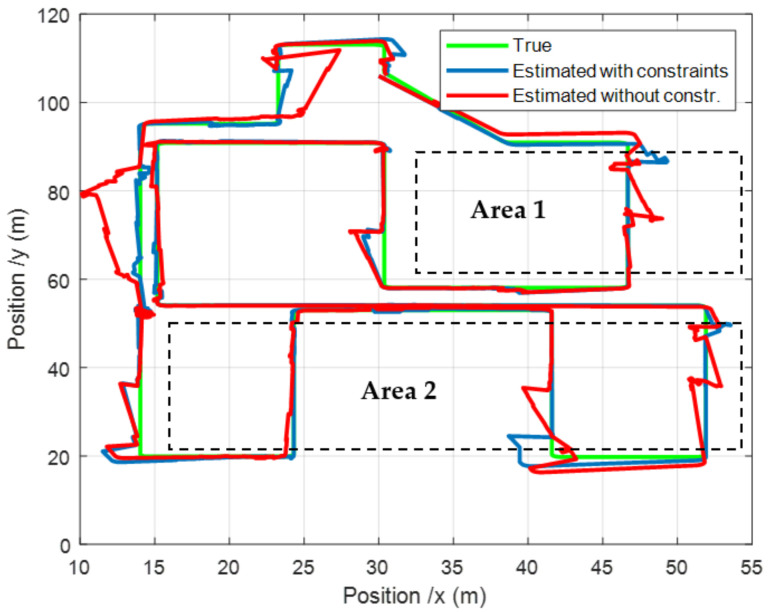
Comparison of the 2D positions with and without constraint applications.

The localization results are given in Figure 6 for the position with respect to the time and in Figure 7 for the 2D trajectory. 

The same journey was also done five times at different speeds, with three different devices: two barcode readers and one tablet. During these experiments, the RSSI and IMU measurements were sent to a log server through a 4G connection and a 4G Teltonika RUT950 router, to create a database. The constrained and unconstrained LBS algorithms were then applied using this database. The results in terms of RMS (root mean square) are summarized in Table 1. The length of each journey and the total number of RSSI detected during the route are also given for information. 

## 5. Discussion

Let us now discuss about the results presented in the previous section. It can clearly be seen in Figure 7 that taking into account the speed constraint leads to a better tracking of the trajectory. It is true in the storage areas, but it also has a positive impact outside, because the heading estimate is more accurate. This can especially be observed in the last part of the route, when the robot is leaving the area 2. It can also be noticed that the constraint allows for a faster realignment of the estimated trajectory to the real one after each abrupt 90° turn. Such situations are usually tricky because of the difficulty to accurately estimate the heading.

In Figure 6, the impact of the constraint can be seen in the confidence interval ±3×STD. An increase of this interval stands for a localization uncertainty increase, due to the low rate of RSSI detected. It is equivalent to an observability deterioration. Such a phenomenon is from far less important in the constrained case (Figure 6b), more especially along the X axis. It highlights the positive effect of the constraint.

Looking to the figures in Table 1, the first thing to point out is that the worst results were obtained in the shortest journey (the second) and the best ones were in the longest (fourth and fifth). This makes sense because for a given distance, the slower the journey, the longer it is, which involves more RSSI detections. The second thing to notice is that the global RMS over the five journeys and three devices are 1.48 m versus 1.20 m without/with the constraint. The improvement is thus 19%. The best obtained result (device 2, journey 5) is a mean error smaller than 70 cm, which is pretty good considering the warehouse harshness.

**Figure 8:**
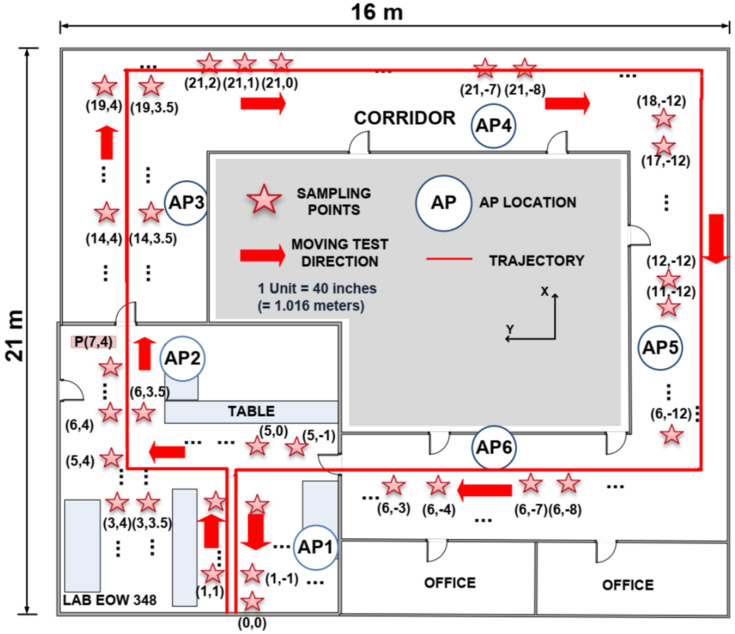
Experiment in the engineering office wing at University of Victoria, considering open datasets [27]. The six access points were located at the AP circles.

To have a point of comparison, our approach was also experimented with the dataset [27] presented in [28]. This dataset included several journeys done by an object (a Nexus 4 running Android 4.4). The data included the real position and the RSSI of the different access points. The route is drawn in Figure 8, extracted from [28].

Unlike in [28], we considered only one MAC address for each access point, i.e., that six RSSI were available at each scan. We also did not consider markers. According to our approach, four areas can be considered, as detailed in Table 2.

The constraint equations suitable with these areas are:

(26) $$Dx_{k}+\zeta_{k}=0\;\;\mathrm{with}:\;D=\left\{\begin{array}{c} \left[0\;0\;0\;1\;0\right]\;\mathrm{for}\;\mathrm{areas}\;1\;\mathrm{and}\;3\\ \left[0\;0\;1\;0\;0\right]\;\mathrm{for}\;\mathrm{areas}\;2\;\mathrm{and}\;4 \end{array}\right..$$

**Figure 9:**
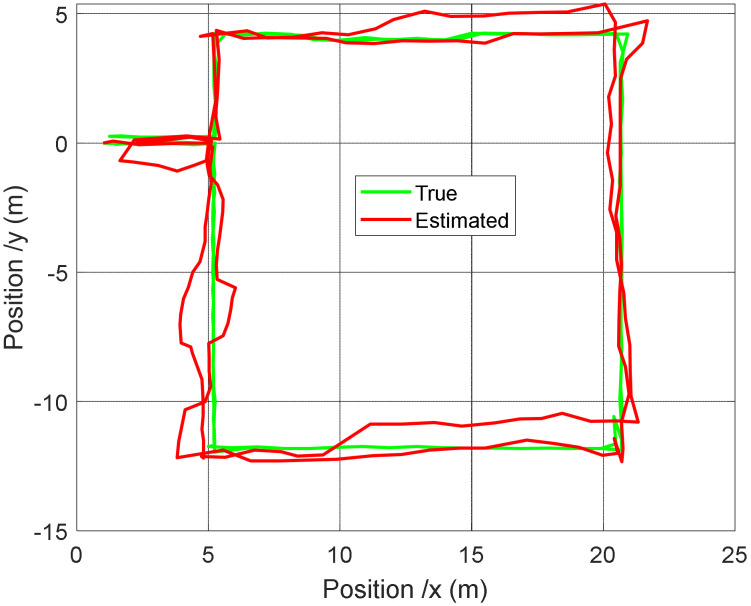
Real (ground truth) trajectory and the estimated one with our approach.

The results in Table 3 highlight that even if our approach is slightly less efficient than the Soft Range Limited K-Nearest Neighbors (SRL-KNN) algorithm introduced in [27], it has smaller average errors than all of the other methods. The estimated 2D trajectory is plot and compared with the real one in Figure 9.

## 6. Conclusions

In this paper, a constrained Kalman filter for Wi-Fi-based indoor localization was developed. This approach is suitable when the space organization is flexible, as in warehouses. The constraint is about the object speed and only applies in some areas of the warehouse. The object to track can be spun around, for example a barcode reader in a hand. The proposed approach was experimented with a robot and three devices on five different journeys. The results highlight that the proposed approach improves the localization accuracy by 19%. As an outlook of this work, the proposed approach can be extended to the 3D case. The use of an estimator such as the UKF and nonlinear smoothing will also be considered. Finally, the regional classifications analysis is another prospect of this work.

## Figures and Tables

**Table 1:** RMS localization errors (in m) with and without constraint applications.

	Device 1	Device 2	Device 3
Journey 1 Length: 8′09″	Nbr of RSSI detect.: 105 RMS unconstr.: 1.86 RMS constrained: 1.05	Nbr of RSSI detect.: 155 RMS unconstr.: 1.31 RMS constrained: 1.28	Nbr of RSSI detect.: 107 RMS unconstr.: 1.53 RMS constrained: 1.36
Journey 2 Length: 6′34″	Nbr of RSSI detect.: 127 RMS unconstr.: 1.87 RMS constrained: 1.43	Nbr of RSSI detect.: 94 RMS unconstr.: 2.26 RMS constrained: 1.68	Nbr of RSSI detect.: 114 RMS unconstr.: 1.59 RMS constrained: 1.58
Journey 3 Length: 8′25″	Nbr of RSSI detect.: 115 RMS unconstr.: 1.76 RMS constrained: 1.04	Nbr of RSSI detect.: 151 RMS unconstr.: 0.93 RMS constrained: 0.89	Nbr of RSSI detect.: 244 RMS unconstr.: 1.01 RMS constrained: 0.94
Journey 4 Length: 10′38″	Nbr of RSSI detect.: 90 RMS unconstr.: 1.43 RMS constrained: 1.01	Nbr of RSSI detect.: 105 RMS unconstr.: 1.23 RMS constrained: 0.96	Nbr of RSSI detect.: 142 RMS unconstr.: 1.56 RMS constrained: 1.38
Journey 5 Length: 10′34″	Nbr of RSSI detect.: 150 RMS unconstr.: 1.31 RMS constrained: 1.12	Nbr of RSSI detect.: 123 RMS unconstr.: 0.77 RMS constrained: 0.66	Nbr of RSSI detect.: 119 RMS unconstr.: 0.89 RMS constrained: 0.75

**Table 2:** Constraint areas in Figure 8.

	Area 1 (Including AP3)	Area 2 (Including AP4)	Area 3 (Including AP5)	Area 4 (Including AP6)
$X_{\min}$	6 m	16.5 m	6 m	0 m
$X_{\max}$	16.5 m	21 m	16.5 m	6 m
$Y_{\min}$	2 m	−11 m	−14 m	−11 m
$Y_{\max}$	4 m	2 m	−11 m	2 m

**Table 3:** Comparison of the average errors.

Our approach: 0.89 m	SRL-KNN [28]: 0.66 m to 1.2 m	STI-WKNN [29]: 1.09 m
Spearman rank [30]: 1.45 m	Kernel method [31]: 1.07 m	Kalman [32]: 0.96 m

## Data Availability

This work utilized part of the open data [27], which can be found at: https://ieee-dataport.org/open-access/wifi-rssi-indoor-localization (accessed on 21 November 2021).
